# Deletion of inositol polyphosphate 4-phosphatase type-II B affects spermatogenesis in mice

**DOI:** 10.1371/journal.pone.0233163

**Published:** 2020-05-15

**Authors:** Yasemin Ceyhan, Manqi Zhang, Jingtao Guo, Carlos G. Sandoval, Jean Vacher, Elena M. Kaftanovskaya, Alexander I. Agoulnik, Irina U. Agoulnik

**Affiliations:** 1 Department of Human and Molecular Genetics, Herbert Wertheim College of Medicine, Florida International University, Miami, FL, United States of America; 2 Division of Medical Oncology, Department of Medicine, Duke University, Durham, NC, United States of America; 3 Department of Oncological Sciences and Huntsman Cancer Institute, Howard Hughes Medical Institute, University of Utah School of Medicine, Salt Lake City, UT, United States of America; 4 Department of Surgery (Andrology/Urology), Center for Reconstructive Urology and Men’s Health, University of Utah Health Sciences Center, Salt Lake City, UT, United States of America; 5 Department of Medicine, Institut de Recherches Cliniques de Montréal, Université de Montréal, Montréal, Québec, Canada; 6 Biomolecular Science Institute, Florida International University, Miami, FL, United States of America; Universite Clermont Auvergne, FRANCE

## Abstract

Inositol polyphosphate-4-phosphatase type II (INPP4B) is a dual-specificity phosphatase that acts as a tumor suppressor in multiple cancers. INPP4B dephosphorylates phospholipids at the 4th position of the inositol ring and inhibits AKT and PKC signaling by hydrolyzing of PI(3,4)P2 and PI(4,5)P2, respectively. INPP4B protein phosphatase targets include phospho-tyrosines on Akt and phospho-serine and phospho-threonine on PTEN. INPP4B is highly expressed in testes, suggesting its role in testes development and physiology. The objective of this study was to determine whether *Inpp4b* deletion impacts testicular function in mice. In testis, *Inpp4b* expression was the highest in postmeiotic germ cells in both mice and men. The testes of *Inpp4b* knockout male mice were significantly smaller compared to the testes of wild-type (WT) males. *Inpp4b*^*-/-*^ males produced fewer mature sperm cells compared to WT, and this difference increased with age and high fat diet (HFD). Reduction in early steroidogenic enzymes and luteinizing hormone (LH) receptor gene expression was detected, although androgen receptor (AR) protein level was similar in WT and *Inpp4b*^*-/-*^ testes. Germ cell apoptosis was significantly increased in the knockout mice, while expression of meiotic marker γH2A.X was decreased. Our data demonstrate that INPP4B plays a role in maintenance of male germ cell differentiation and protects testis functions against deleterious effects of aging and high fat diet.

## Introduction

Male infertility accounts for approximately half of failed conceptions after 12 or more months of regular, unprotected sexual intercourse [1]. A substantial portion of men have suboptimal sperm parameters such as low sperm count, poor mobility or abnormal morphology, which can all contribute to infertility. Testicular abnormalities, aberrant hormone production, and failed spermatogenesis are the most common causes of congenital male infertility [2, 3]. In addition to genetic factors, environmental factors also play an important role in male infertility. One of the most studied environmental factors affecting fertility is obesity [4, 5]. Obesity is correlated with a reduction in sperm quality and low rates of pregnancy [6, 7]. However, some obese patients do not develop these defects, suggesting the existence of molecular mechanisms protecting testicular function against environmental insults.

The phosphatidylinositol signaling pathway is critical to the regulation of a variety of cellular activities including cell metabolism, morphogenesis, cell cycle, cytoskeletal organization, cell polarity, and membrane trafficking. The main mechanism of regulation in this pathway relies on the controlled phosphorylation and de-phosphorylation of specific membrane bound lipids, phosphatidylinositol polyphosphates (PIPs), at the 3-, 4-, and 5-positions of the inositol ring [8]. Two best described phosphatases, phosphatase and tensin homolog (PTEN) and inositol polyphosphate 4-phosphatase II (INPP4B), dephosphorylate PIPs at the 3- and 4-inositol positions respectively, inhibiting the Akt signaling pathway. Both PTEN and INPP4B are widely expressed and function as tumor suppressors in multiple cancers. Recent data in fruit flies, frogs, mice and other species indicate the importance of kinases and phosphatases in the PIP pathway in the development of male germ cells [9]. INPP4B is a cytosolic membrane dual specificity phosphatase that dephosphorylates both phospholipids and phosphoproteins. It possesses an N-terminal, C2 lipid-binding domain, an internal NHR2 (Nervy Homology 2) domain, and the conserved dual phosphatase motif, CX5R, within the C-terminal phosphatase domain [10, 11]. Our lab and others have shown that INPP4B participates in a variety of signaling pathways including PI3K/Akt and PKC. Notably, the loss of INPP4B correlates with poor prognosis in human cancer, including cancers of the male reproductive system [12–14]. However, the INPP4B role in healthy organs remains largely unknown.

Recent studies confirmed that INPP4B and PTEN are highly expressed in the adult human [15, 16] and mouse [17, 18] testis. Here we report the cell specific expression pattern of INPP4B in human and mouse testis and describe morphological and functional changes in mouse testis lacking functional INPP4B. We show that INPP4B is highly expressed in postmeiotic germ cells. Analysis of circulating hormones revealed reduced testosterone and LH concentrations in the serum of *Inpp4b*-deficient males. This reduction was associated with decreased expression of critical steroidogenic enzymes, reduced testes size, and decreased sperm production that worsens with age. There was a higher rate of apoptosis and a decrease in the expression of meiosis marker γH2A.X in *Inpp4b*^*-/-*^ testis. A high fat diet exacerbated the effects of INPP4B loss in testicular function. The results suggest an important role for INPP4B in testicular physiology.

## Materials and methods

### Analysis of single cell RNA-sequencing, clustering and gene ontology (GO) pathway analysis

RNA sequencing and gene clustering was performed using Seurat and previously reported data [19]. The Gene Ontology pathway analysis of genes positively correlated with INPP4B expression in testis was performed using DAVID bioinformatics functional annotation tool using data for all testicular cells for comparison [20, 21]. Since single cell RNA data results in a high dropout rate (~50%), R = 0.4 was used for cutoff. The adjusted p values less than 0.05 were accepted as significant. To detect the expression of a gene in various human testicular cells, the Human Testis Atlas Browser (https://humantestisatlas.shinyapps.io/humantestisatlas1/) was used [19].

### Animal studies

Mice were maintained at the AAALAC accredited animal facility at Florida International University and all experimental protocols were performed in accordance with the regulations of the Institutional Animal Care and Use Committees at FIU and the National Academy of Science Guide for Care and Use of Laboratory Animals. The Institutional Animal Care and Use Committees at Florida International University approved this research, protocol AN18-055.

Generation of conventional knockout *Inpp4b*^*-/-*^ [22, 23] and cryptorchid *Rxfp2*^*-/-*^ [24] mouse models were described previously. Mice with the *Inpp4b* knockout allele were backcrossed to FVB/N inbred strain for 4 generations and then intercrossed to obtain *Inpp4b*^*-/-*^ homozygotes in order to decrease genomic background variability. Mice were fed with low fat diet (LFD) with 12.9% fat, 63.8% carbohydrate, and 23.2% protein (total 13.6 kJ/g) (LabDiet 5V75, St. Louis, MO) or high fat diet (HFD) with 59.4% fat, 25.7% carbohydrate, and 14.9% protein (total 22.8 kJ/g) content (TestDiet 58R3, St. Louis, MO) [25]. For the HFD group, the females were on HFD for a month prior, during the pregnancy, and after delivery until weaning and the pups were on HFD from weaning until the euthanasia [25]. It was shown that under this protocol, male pups displayed more drastic changes in the urogenital system. Age-matched WT and *Inpp4b* ^-/-^ male mice were used in our studies at 2, 3, 4 and 6 months of age. Mice were euthanized by isoflurane (Patterson Veterinary, Greeley, CO) overexposure and testes and seminal vesicles were dissected and weighed.

Sperm count was performed as described by Huang *et al* [24], with minor modification. Briefly, sperm was released from the cauda epididymis into PBS, then diluted at a 1:5 ratio with distilled water, loaded into a hemocytometer and counted manually under the microscope by two investigators.

### Hormone measurement

Testosterone (T) and LH levels were determined in male blood serum. The mice were euthanized, and blood collected from 3-month old mice, with 6 males per group. The serum was collected after centrifugation at 3000 × *g* for 15 min and frozen until hormonal analysis. Testosterone and LH levels were determined in the University of Virginia Center for Research at the Reproduction Ligand Assay and Analysis Core (University of Virginia, Charlottesville, VA) using mouse/rat testosterone ELISA (IBL America, Minneapolis, MN) and RIA (in house protocol) assays respectively.

### Gene expression analysis

mRNA samples were isolated from mouse testes using Tri-Reagent (Molecular Research Center, Cincinnati, OH) and reverse transcribed using the Verso cDNA synthesis Kit (Thermo Fisher Scientific, Waltham, MA). For quantitative PCR, primers were designed using online software (Roche, Basel, Switzerland) and probes were purchased from Universal ProbeLibrary (Roche). Roche 480 LightCycler (Roche) was used for probe-based real-time PCR. GoTaq qPCR master mix (Promega, Madison, WI) was used for experiments using BRYT dye on the Mastercycler RealPlex^2^ system (Eppendorf, Westbury, NY). The relative fold change in gene mRNA level was calculated by the comparative cycle threshold (2^–ΔΔ*C*t^) method using 18S rRNA for normalization of the expression data. Primer sequences are shown in Table 1. The number of animals used in each group is shown in figure legends.

**Table 1:** Primers used for qRT-PCR.

Gene	Forward primer	Reverse primer	Probe
*18S*	gcaattattccccatgaacg	gggacttaatcaacgcaagc	48
*Inpp4b*	tgaccctgaggacattcagtt	attccaactgtggctcgttc	89
*Pten*	aggcacaagaggccctagat	ctgactgggaattgtgactcc	60
*Tnfα*	ttgtcttaataacgctgatttggt	gggagcagaggttcagtgat	64
*Il1b*	caggcaggcagtatcactca	tgtcctcatcctggaaggtc	76
*Il6*	gctaccaaactggatataatcagga	ccaggtagctatggtactccagaa	6
*Hsd17b3*	tggtcccctataacagagcttca	gaaaagtcctgcccatttgt	5
*Hsd3b6*	accatccttccacagttctagc	acagtgaccctggagatggt	95
*Cyp11a1*	cctgagaaccccatcctctt	agtgttgtcttttctggtcacg	11
*Cyp17a1*	catcccacacaaggctaaca	cagtgcccagagattgatga	BRYT
*Cyp19*	cgaagcagcaatcctgaaggag	ccaagtccacaacaggctggta	BRYT
*Star*	ggaagtccctccaagactaaac	tggttgatgattgtcttcgg	BRYT
*Lhcgr*	caggaatttgccgaagaaag	tggagtgtcttgggtgaaca	BRYT
*Srd5a1*	gatggtgggctcttcctacg	aaaaccagcgtcctttgcac	BRYT
*Nr5a1*	gtgcatggtctttaaggagctgg	ggatgctgtcttccttgccgta	BRYT

### Western blotting

Testes from WT, *Inpp4b* ^*-/-*^ and cryptorchid *Rxfp2* ^*-/-*^ mice were homogenized with a glass tissue grinder in ice-cold RIPA buffer supplemented with protease and phosphatase inhibitors [23]. The lysates were diluted tenfold and 20–30 μg of protein were resolved on SDS-PAGE and transferred to PVDF membrane. For immunoblotting, rabbit polyclonal primary antibody against AR (1:1000 dilution, catalog number 06–680, Millipore, Carlsbad, CA) and mouse monoclonal β-tubulin (1:5000, #05–661, Millipore) were used. Signal was visualized using ImageQuant LAS 500 imaging system (GE Healthcare Life Sciences, Marlborough, MA) and quantitative analysis was performed with ImageQuant TL software.

### Propidium iodide staining and flow cytometry

Testis digestion and flow cytometry experiments were performed as previously described [26]. Six-month old WT and *Inpp4b*^*-/-*^ mice were used (n = 4 for each group). Seminiferous tubules were washed with 1X HBSS, digested with collagenase and trypsin, and filtered. Next, cells were washed with 0.1% BSA in PBS, centrifuged at 400 g for 2 minutes, counted, and resuspended at 2x10^6^ cells/ml. Cells were fixed in 70% ethanol and stained for 30 minutes with 25 μg/ml of propidium iodide dissolved in 0.1% BSA in PBS. Analysis was performed using Accuri C6 flow cytometer (Becton-Dickinson, Franklin Lakes, NJ).

### Histology and immunohistochemistry (IHC)

Testis samples were fixed in 4% PFA overnight and embedded in paraffin. The embedded tissue was sectioned at 4.5 μm. H&E staining and IHC were performed as previously described [27]. Rabbit polyclonal antibodies to INPP4B (1:150 dilution, #8450, Cell Signaling, Danvers, MA) and γH2A.X (1:700, #2577, Cell Signaling, Danvers, MA) were used as primary antibodies and sections were counterstained with hematoxylin (Millipore). For TUNEL assay, ApopTag Plus Peroxidase *In Situ* Apoptosis Detection Kit (Millipore) was used following manufacturer protocol. Three mice were analyzed for each group and minimum ten circular tubules were counted per mouse. The images were captured using a Carl Zeiss Axio A1 microscope with an AxioCam MRc5 CCD camera (Carl Zeiss, New York, NY).

### Statistical analysis

Student’s t-test for two groups, one-way ANOVA, and two-way ANOVA for more than two groups were used to assess the significance of differences using Prism 7.0 software (GraphPad Software, La Jolla, CA). All data are presented as mean ± SEM. p values less than 0.05 (p<0.05) were accepted as significant. The number of samples analyzed is shown in figure legends.

## Results

### INPP4B expression is highest in post-meiotic germ cells

**Fig 1:**
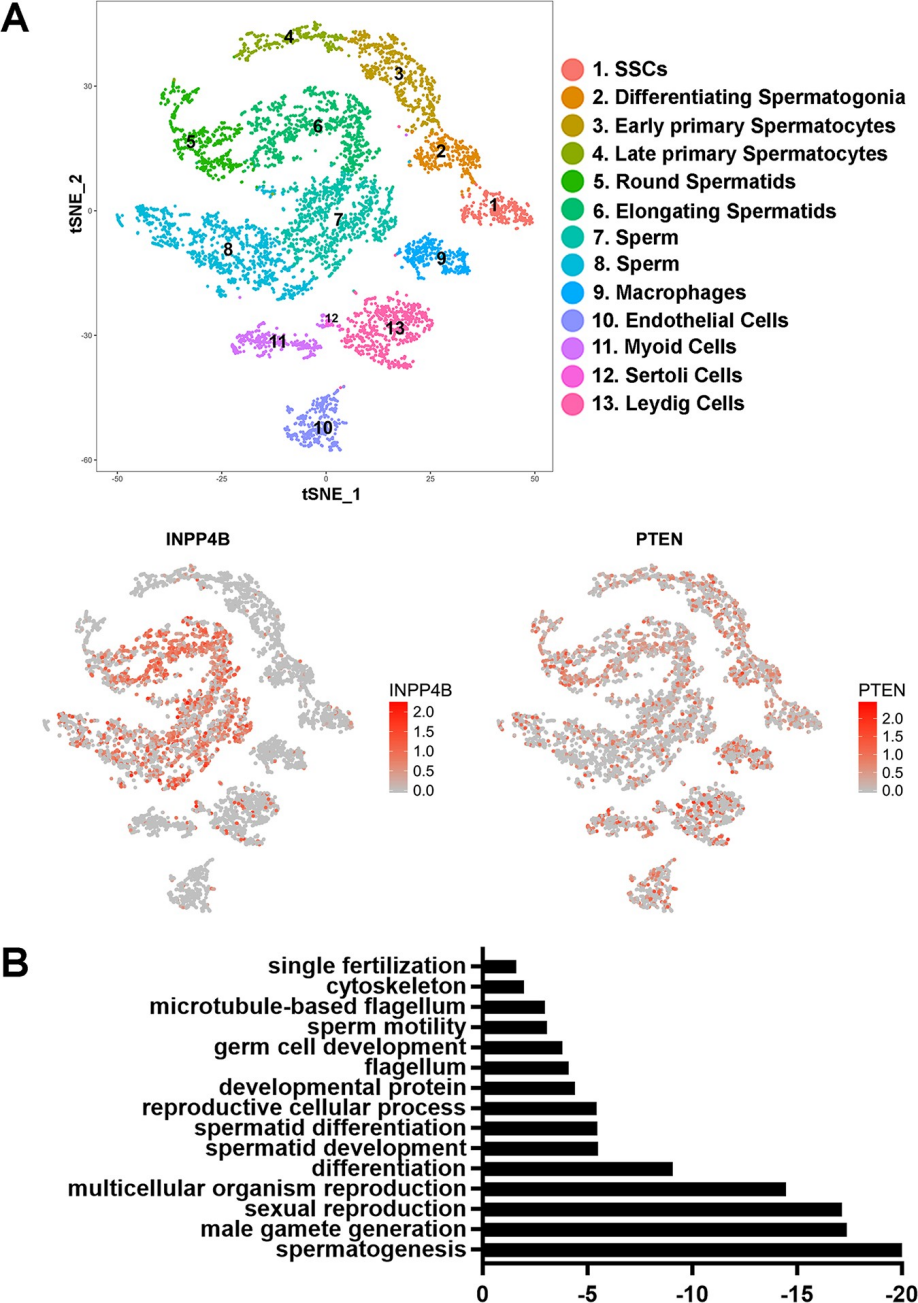
INPP4B is preferentially expressed in the post-meiotic germ cells in mouse testis. A) INPP4B and PTEN expression map in different compartments of testis, obtained by the analysis of single cell RNA-Seq data. The map for the different cell fractions was adapted from [19]. B) Gene ontology pathways regulated by top 222 genes upregulated in INPP4B-positive germ cells. The vertical axis shows the top 15 pathways that correlate with high INPP4B levels and the horizontal axis represents the logarithmic scale of p values. The data was obtained by analyzing single cell RNA-Seq data sets [19].

To understand the function of *INPP4B* in the testes, we analyzed the expression pattern of *INPP4B* in various testicular cell populations. Using a previously generated transcriptional cell atlas [19] derived from human testicular single-cell RNA sequencing, we detected the highest level of *INPP4B* expression in the round and elongating spermatids and in differentiating sperm (Fig 1A). A lower level of expression was also present in Leydig and Sertoli cells. Interestingly, *PTEN* was expressed ubiquitously in stromal and germ cells of the testis (Fig 1A). Clustering gene analysis and gene ontology analysis showed that the top 222 genes upregulated with correlation coefficient cut-off value 0.4 in *INPP4B*-positive cells are involved in fertilization, reproductive development, cellular signaling pathways and various stages of spermatogenesis (Fig 1B). Thus, *INPP4B*-positive cells represent germ rather than somatic cell populations in testis.

**Fig 2:**
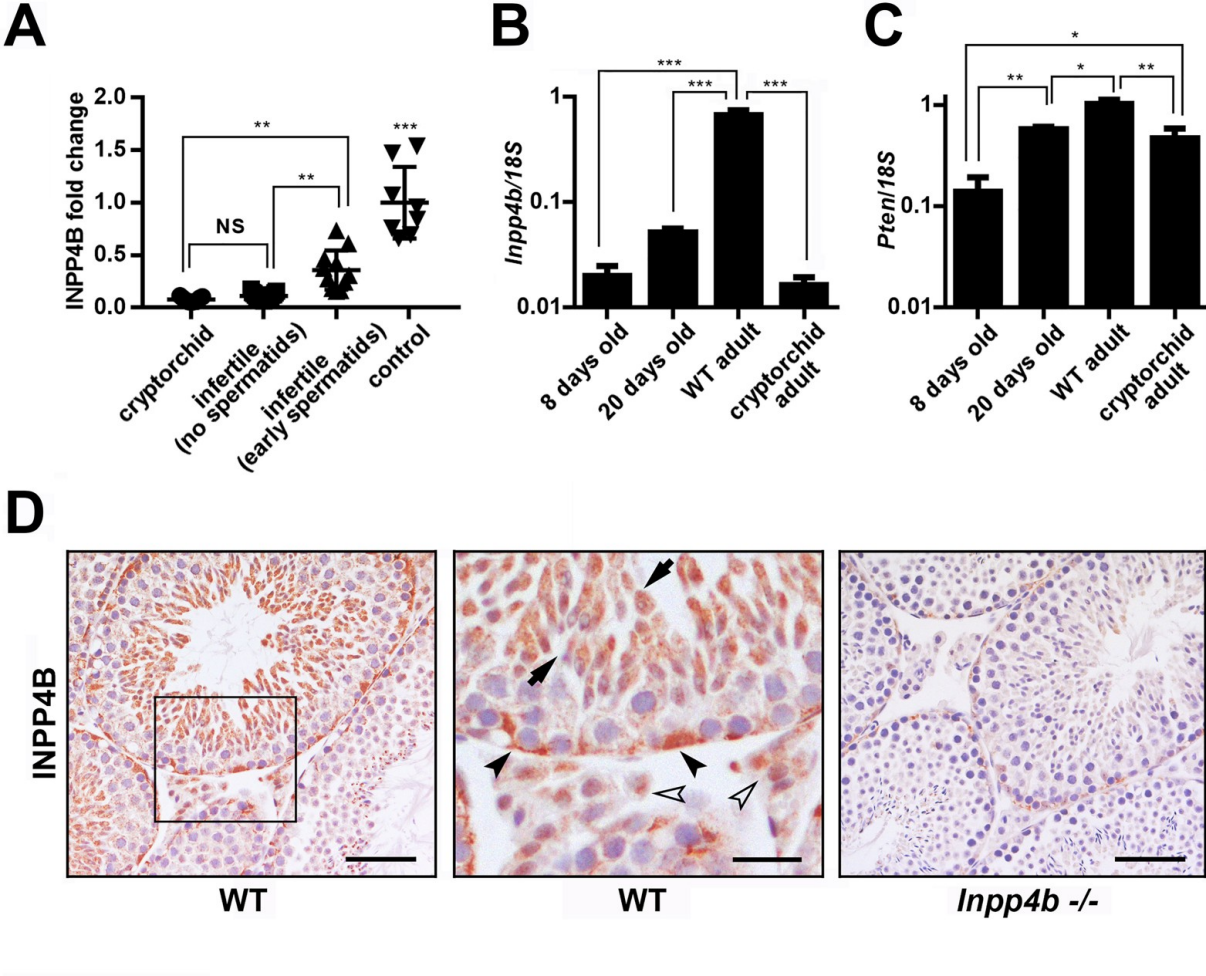
INPP4B and PTEN gene expression during spermatogenesis. A) Expression analysis of *INPP4B* in cryptorchid, infertile patients without spermatids, infertile patients with spermatids and control groups using microarray data from Chalmel et al [28]. The control group has higher *INPP4B* expression compared to all other groups. **p<0.01, ***p<0.001. B) *Inpp4b* and C) *Pten* expression in postnatal mouse testes analyzed by qRT-PCR. RNA was extracted from 8-day old (n = 10), 20-day old (n = 6), WT adult (n = 6), and cryptorchid adult (n = 8) whole testis. Gene expression was normalized to *18S*. Data shown as mean ± SEM. Statistical analysis was performed using 1-way ANOVA. *p<0.05, **p<0.01, ***p<0.001. D) INPP4B IHC of 2-month old mouse testes. Testis sections from age-matched WT (left panel) and *Inpp4b*^**-/-**^ males (right panel) were stained for INPP4B and counterstained with hematoxylin. The middle panel represents a magnified section of WT testis. Elongated spermatids showed by arrows, Leydig and Sertoli cells showed by white and black arrowheads respectively. No staining was detected in *Inpp4b*-deficient testis sections. Scale bars represent 100 μm for right and left images and 20 μm for the middle image.

We next used the microarray data from Chalmel et al. [28], and analyzed *INPP4B* expression in the testes of men with cryptorchidism or infertility and compared it to *INPP4B* expression in healthy adult individuals. We divided the infertile population into two groups, those who had spermatids present in the seminiferous tubules and those who did not. Among all groups, *INPP4B* expression was highest in healthy controls and it was significantly decreased in cryptorchid testes. Within the infertile population, *INPP4B* expression was significantly lower in the group lacking spermatids (Fig 2A).

The first cycle of spermatogenesis in mice is synchronized over seminiferous tubules. This timely process allows the comparison of the gene expression within distinct stages of germ cell differentiation. We compared *Inpp4b* and *Pten* expression in the testes at three important time-points during germ cell development: day 8, when the testes contain pre-meiotic diploid differentiating germ cells up to the type B spermatogonia stage; day 20, when the germ cells are differentiated into round spermatids; and after day 35, when testes contain all stages of germ cells [29]. We also analyzed adult testes from 2-month old *Rxfp2*^-/-^ males with high intraabdominal cryptorchidism, which lack all stages of spermatogenesis past the spermatogonial cells, with only a few early spermatocytes present [26]. When compared to adult WT mice, *Inpp4b* expression in these mutants was significantly lower in the testes of 8- and 20-day old mice, and cryptorchid adult mice (Fig 2B). Cryptorchid testes show a somewhat lower level of expression compared to 20-day old WT testis (Fig 2B), although this difference does not reach statistical significance (p = 0.868). Thus, as in human, in mouse germ cells *Inpp4b* expression was highest during the late stages of spermatogenesis. The variation of PTEN expression was significantly less pronounced in the same groups (Fig 2C). IHC staining indicated robust expression of INPP4B was present in elongated spermatids and lower expression was observed in round spermatids, Leydig and Sertoli cells in WT mouse testes (Fig 2D).

### Testis weight and sperm counts are decreased in *Inpp4b*^*-/-*^ males

**Fig 3:**
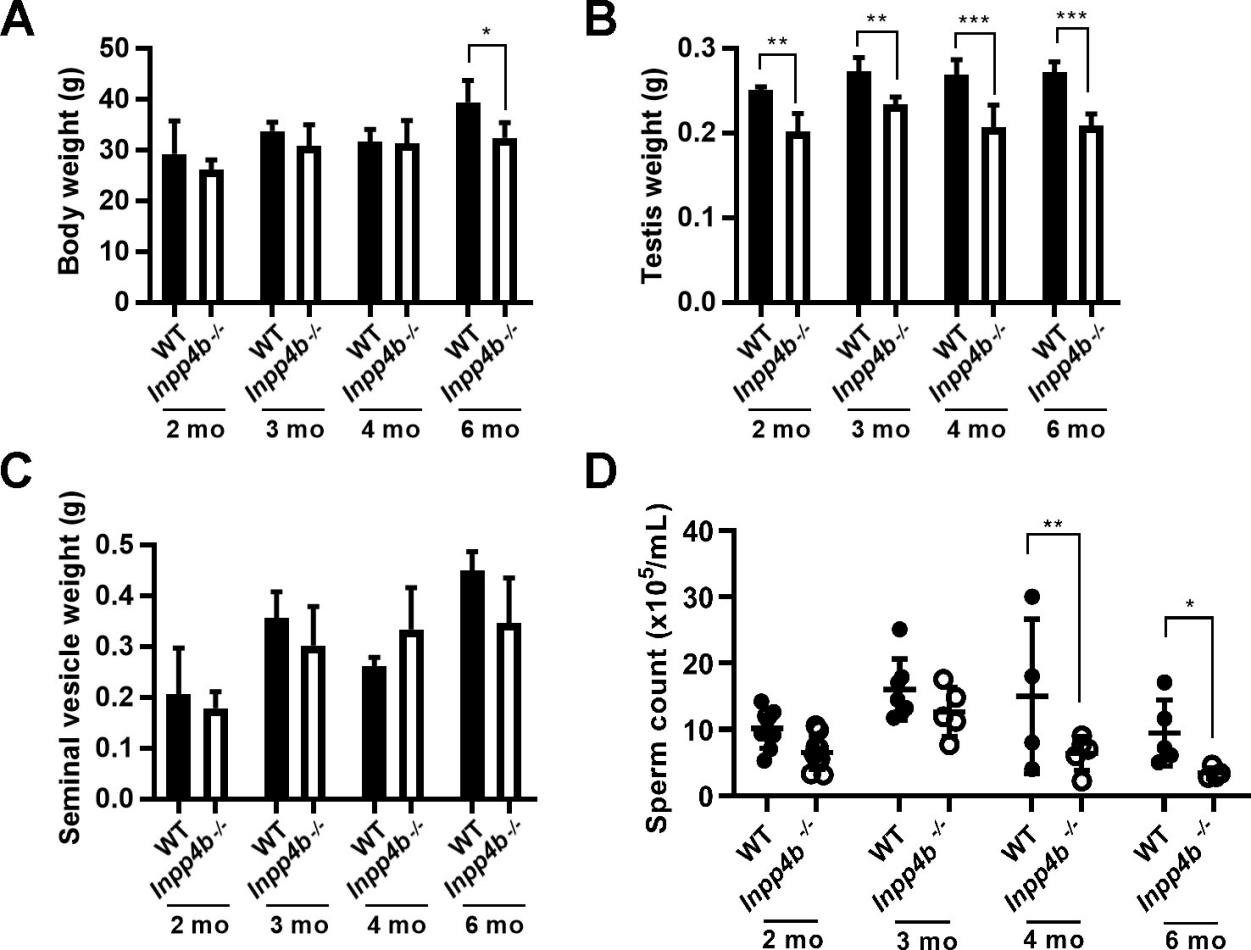
Testicular weight and epididymal sperm counts in *Inpp4b* ^*-/-*^ mice. A) Body weights for 2-, 3-, 4-, and 6-month old mice. B) Testes weights of 2-, 3-, 4- and 6-month old mice. C) Seminal vesicle weights for 2-, 3-, 4- and 6-month old mice. D) Epididymal sperm count for 2-, 3-, 4- and 6-month old mice. *p<0.05, **p<0.01, ***p<0.001; n>4/group. Data shown as mean ± SEM. Statistical analysis was performed using 2-way ANOVA.

To characterize the effect of INPP4B loss, we measured body, testes, and seminal vesicle weight and the epididymal sperm count of 2-, 3-, 4- and 6-month old WT and *Inpp4b*^*-/-*^ mice. Body weight remained comparable among age groups until 6-months of age, when *Inpp4b*^*-/-*^ mice weighed slightly less (Fig 3A). In *Inpp4b*^*-/-*^ males, testes weight was consistently smaller when compared to WT controls, and this difference increased with age (Fig 3B). There were no statistically significant differences in seminal vesicle weight between WT and *Inpp4b*^*-/-*^ mice at any age (Fig 3C) or visible changes in mutant epididymides. Epididymal sperm counts were higher in the WT groups. The difference was not statistically significant between 2-month old and 3-month old males, but it became significant in 4- and 6-month old groups. (Fig 3D). Additionally, there were no differences in the diameter of the seminiferous tubules between WT and *Inpp4b*^*-/-*^ testes at any age group (S1 Fig).

### INPP4B deficiency causes a shift from haploid to diploid cell population in testis

**Fig 4:**
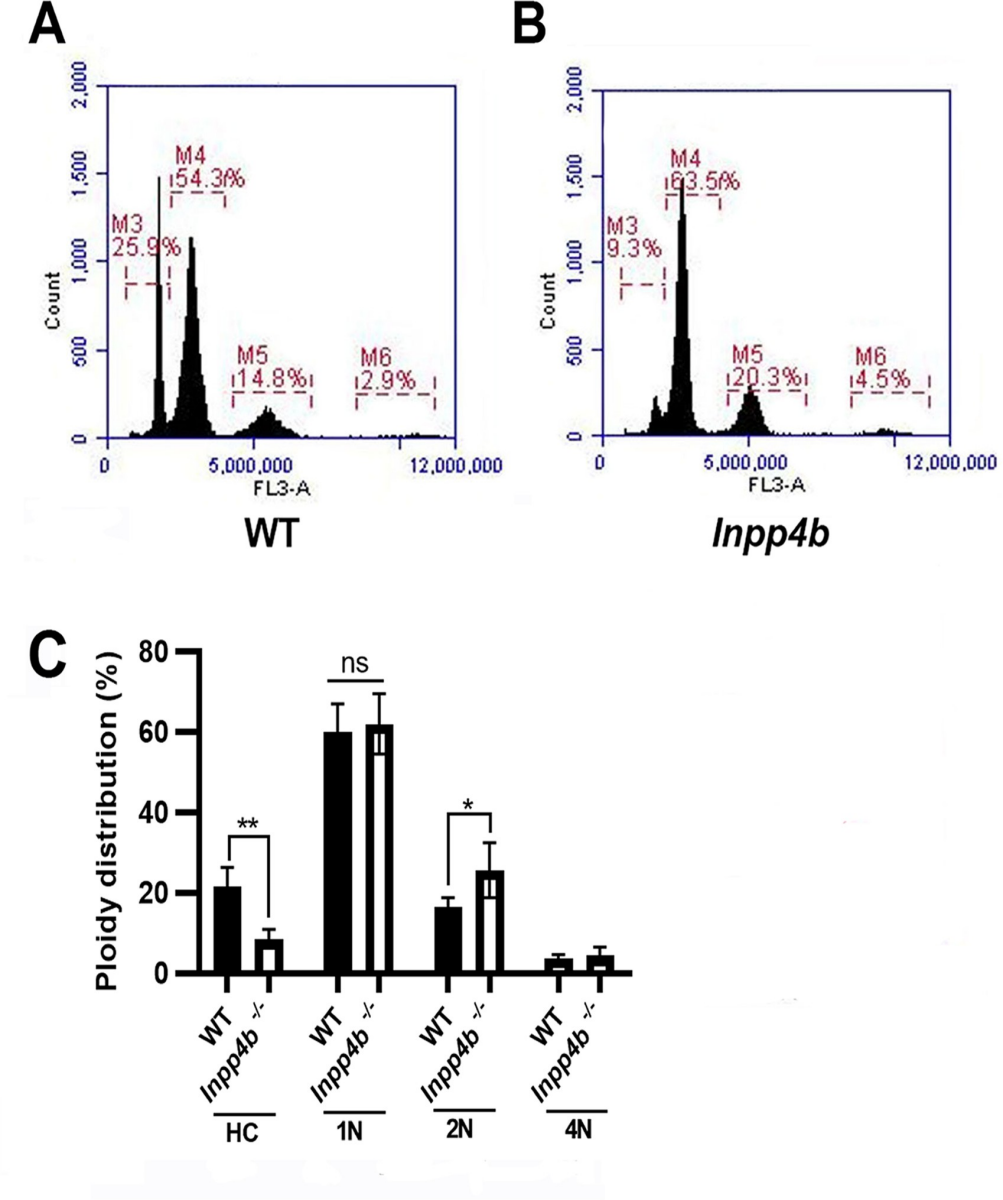
Flow cytometry analysis of testes from WT and *Inpp4b*^-/-^ mice. Flow cytometry histograms of A) wild type B) *Inpp4b*^*-/-*^ testes. Testicular cells have four cell populations distinguished by DNA intensity on the Y axis: M3 (1N, haploid, elongated spermatids), M4 (1N, haploid, round spermatids), M5 (2N, diploid), and M6 (4N, quadriploid). A representative histogram from a 6-month old male in each group is shown. C) Quantitative analysis of cell populations, n = 4/group (*p<0.05, **p<0.01). Data shown as mean ± SEM. Statistical analysis was performed using 2-way ANOVA.

Since *Inpp4b*^*-/-*^ males had significantly decreased sperm counts in aged groups, we used flow cytometry to evaluate changes in the ploidy of germ cells in 6-month old WT and mutant testes [30, 31]. We compared the percentages of testicular haploid, diploid, and tetraploid populations. In the haploid populations, we examined round (1C) and hypostained (HC) elongated spermatids separately to determine whether the reduction in sperm counts was due to a reduction in meiosis (round spermatids) or spermiogenesis (elongated spermatids) stages (Fig 4A and 4B) [32]. The percentage of elongated spermatids was significantly decreased in *Inpp4b*^*-/-*^ males (WT– 21.8%, *Inpp4b*^*-/-*^– 8.7%, p = 0.0032), whereas the percentage of diploid cells in knockout males was increased (WT– 16.6%, *Inpp4b*^*-/-*^– 25.8%, p = 0.0434). The percentages of round spermatids and tetraploid cells were not statistically different between WT and *Inpp4b*^*-/-*^ groups (Fig 4C).

### High fat diet exacerbates the effects of INPP4B deficiency on testis functions

**Fig 5:**
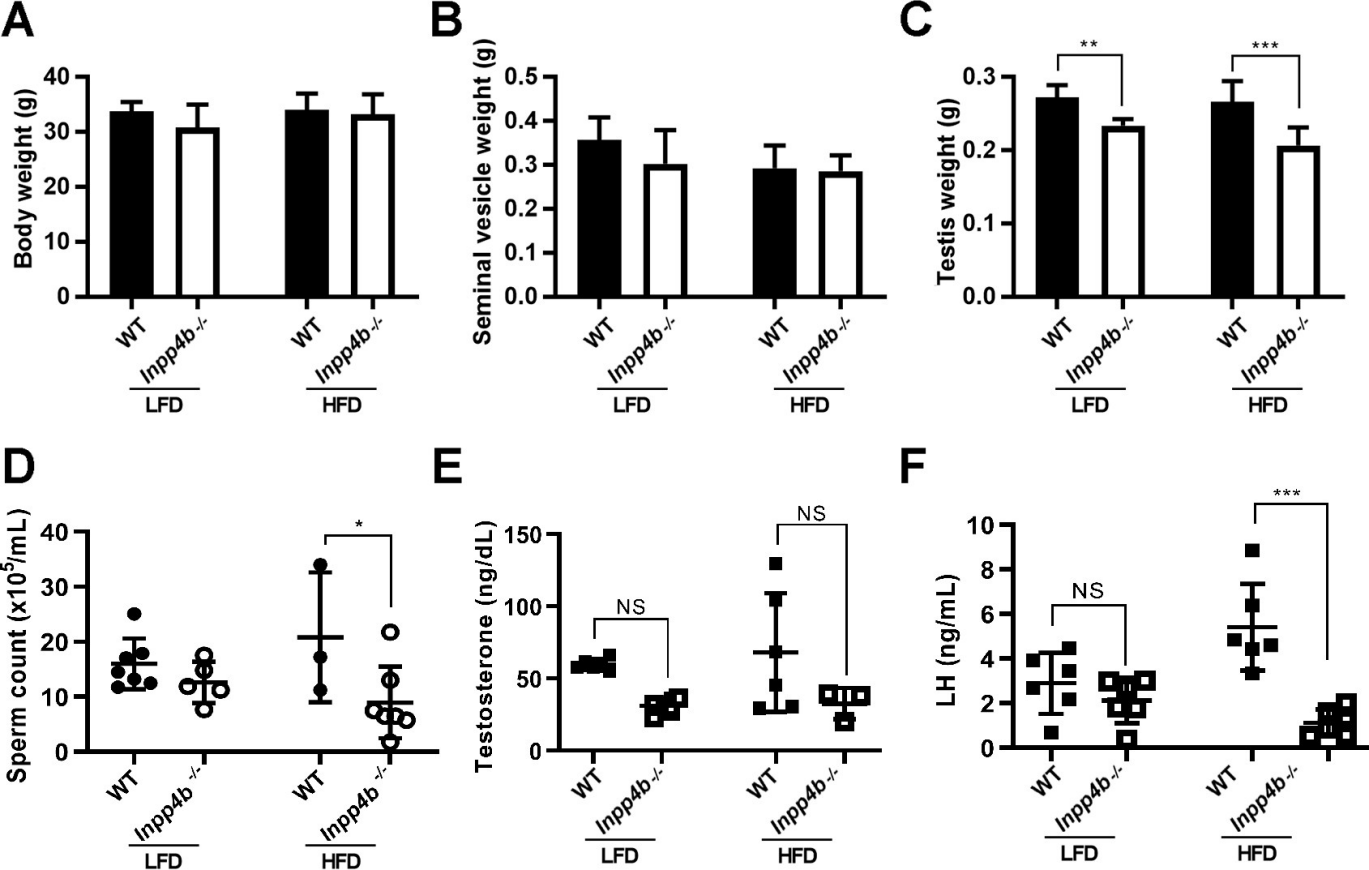
Effect of HFD on testicular phenotype of *Inpp4b*^*-/-*^ males. A) Body weights, B) Seminal vesicle weights, C) Testes weights, D) Epididymal sperm counts, E) Testosterone and F) Luteinizing hormone levels for 3-month old mice on LFD and HFD. *p<0.05, **p<0.01, ***p<0.001; n = 4/group. Data shown as mean ± SEM. Statistical analysis was performed using 2-way ANOVA.

Since INPP4B is a key regulator of PIP substrates that participate in metabolic signaling pathways, we examined if a high fat diet (HFD) can exacerbate the effects of INPP4B loss on spermatogenesis. Body weight and seminal vesicle weights showed no difference when comparing 3-month old *Inpp4b*^*-/-*^ and WT mice fed either diet (Fig 5A and 5B). The testes of *Inpp4b*^*-/-*^ mice weighed significantly less than testes of the WT controls (Fig 5C) and the consumption of a HFD significantly decreased sperm count in *Inpp4b*^*-/-*^ mice when compared to WT (Fig 5D). The LFD group is the same as the 3-month old group used in Fig 3.

Since the hypothalamic-pituitary-gonadal (HPG) hormonal axis has an important role in the regulation of spermatogenesis [33, 34], we compared the levels of testosterone and luteinizing hormone (LH) in the serum of mutant and WT males on a LFD or a HFD. While there was a trend suggesting that *Inpp4b*^*-/-*^ mice produce less testosterone than WT controls, this difference was not statistically significant (Fig 5E). In testis, LH stimulates the production of testosterone in Leydig cells. Notably, in the HFD group, the LH levels were significantly lower in the *Inpp4b*^*-/-*^ group than in WT mice (Fig 5F).

**Fig 6:**
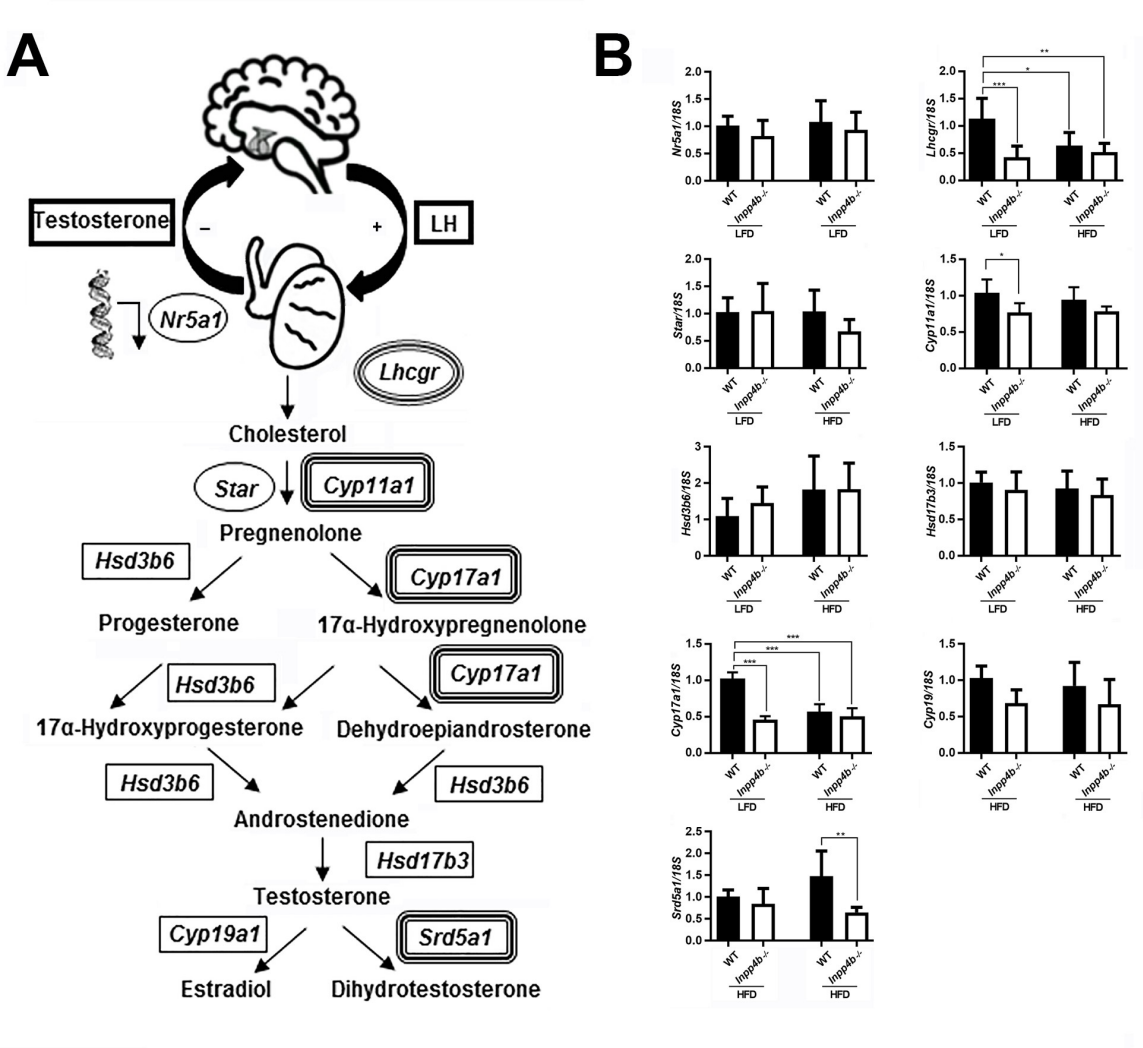
INPP4B deficiency and HFD effect on steroid hormone metabolism. A) Schematic diagram for hypothalamic–pituitary–gonadal hormonal axis and testosterone synthesis in testis. Key enzymes of the steroidogenic pathway are shown; corresponding genes showing different level of expression in mutant animals or on HFD are double-circled. B) Expression of genes in the steroidogenic pathway analyzed by qRT-PCR. The expression level for all genes was normalized to *18S*. *p<0.05, ***p<0.001; n = 7/group. Data shown as mean ± SEM. Statistical analysis was performed using 2-way ANOVA.

Steroidogenic hormone synthesis in testis is controlled by a series of enzymatic steps (Fig 6A). We compared the gene expression of the key steroidogenic enzymes *Cyp11a1*, *Cyp17a1*, *Hsd3b6*, *Hsd17b3*, *Cyp19* and *Srd5a1*, steroidogenic factor-1 *Nr5a1*, the cholesterol transporting protein *Star*, and the LH receptor, *Lhcgr*, in WT and mutant testes in LFD and HFD groups. *Cyp11a1* expression was significantly lower in the *Inpp4b*^*-/-*^ LFD group when compared to WT mice, but this decrease was not significant in HFD groups (Fig 6B). *Cyp17a1* and *Lhcgr* expression was significantly lower in the LFD mutant group and in mice on HFD when compared with the WT LFD group (Fig 6B). *Srd5a1* expression was not changed between LFD groups, but it was significantly decreased in *Inpp4b*^*-/-*^ HFD mice when compared with the WT HFD group. The expression of *Nr5a1*, *Star*, *Hsd3b6*, *Hsd17b3*, and *Cyp19* were not affected by the loss of INPP4B or the diet (Fig 6B).

**Fig 7:**
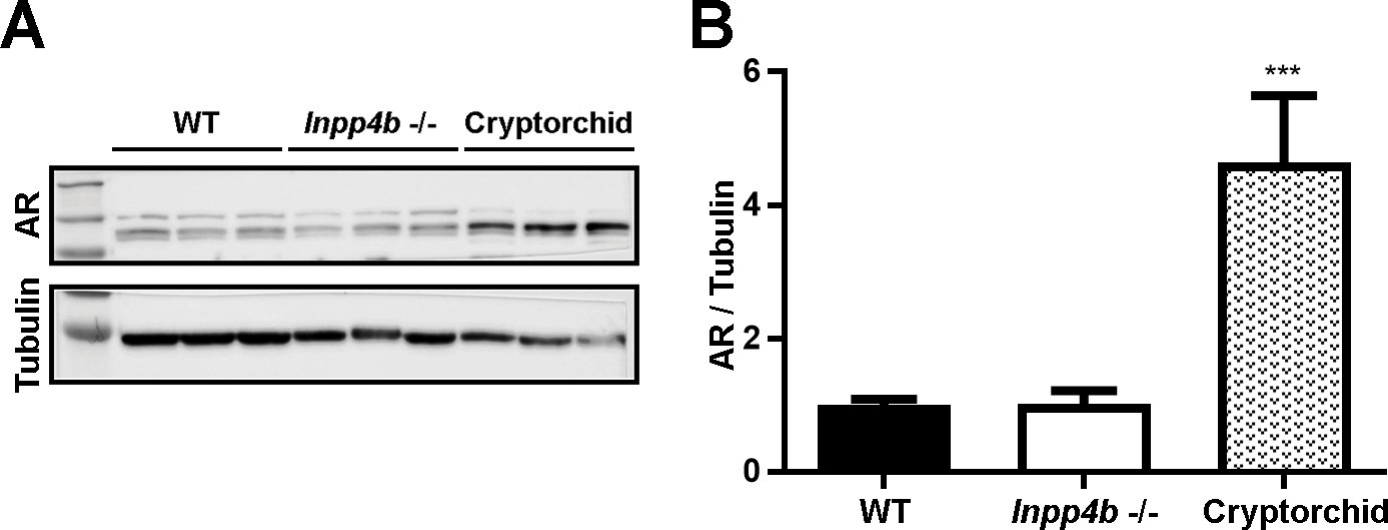
Androgen receptor expression in *Inpp4b*^*-/-*^ testis. A) AR expression in testes of WT, *Inpp4b-/-* and cryptorchid mice was analyzed by Western blot hybridization. B) Densitometric analysis of the Western blots. ***p<0.001; n = 3/group. Data shown as mean ± SEM. Statistical analysis was performed using 2-way ANOVA.

Additionally, there was no difference in testicular androgen receptor (AR) protein levels between WT and *Inpp4b* ^*-/-*^ groups (Fig 7). There was a significantly higher level of AR expression in the cryptorchid testes, likely due to the lack of a germ cell population beyond the spermatogonia stage. As the germ cells do not express AR, cryptorchid testes have a higher proportion of AR-positive stromal cells compared to WT [35].

### Loss of INPP4B increases apoptosis rate in early spermatogenesis

**Fig 8:**
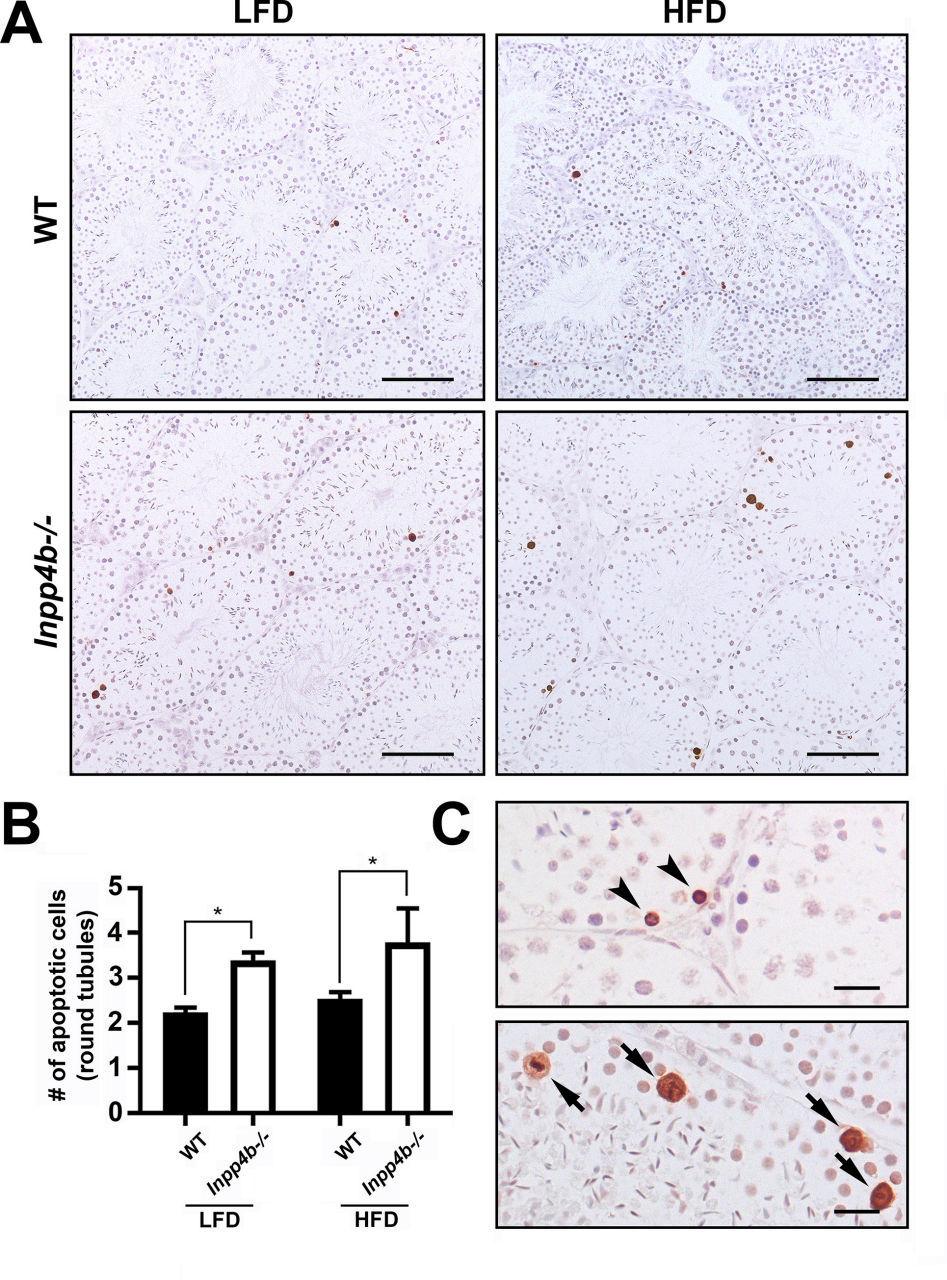
Apoptosis in *Inpp4b*^*-/-*^ testis. A) Increased apoptosis in *Inpp4b*^*-/-*^ testes of 3-month old males on LFD and HFD mice. Cell apoptosis was analyzed by TUNEL assay. A representative image from each group is shown. Scale bar represents 200 μm. B) Apoptotic cells (brown) were counted per field under 20X objective in at least 5 fields in each of 2 sections per animal. *p<0.05; n = 3/group. Data shown as mean ± SEM. Statistical analysis was performed using 2-way ANOVA. C) Magnified sections from *Inpp4b*^**-/-**^ males on HFD. Spermatogonia and cells in meiotic metaphase showed by arrowheads and arrows respectively. Scale bar is 20 μm.

A decrease in the haploid testicular cell population and an increase in diploid cells indicated an abnormality in meiosis or spermiogenesis. We analyzed the rate of apoptosis in testes from 3-month old WT and *Inpp4b*^*-/-*^ testes in LFD and in age-matched HFD groups (Fig 8). The apoptosis rate was significantly higher in the mutant group compared to WT testes, independent of the diet (Fig 8B). Apoptosis in testis was mainly observed in spermatogonia cells and in primary spermatocytes (Fig 8C).

**Fig 9:**
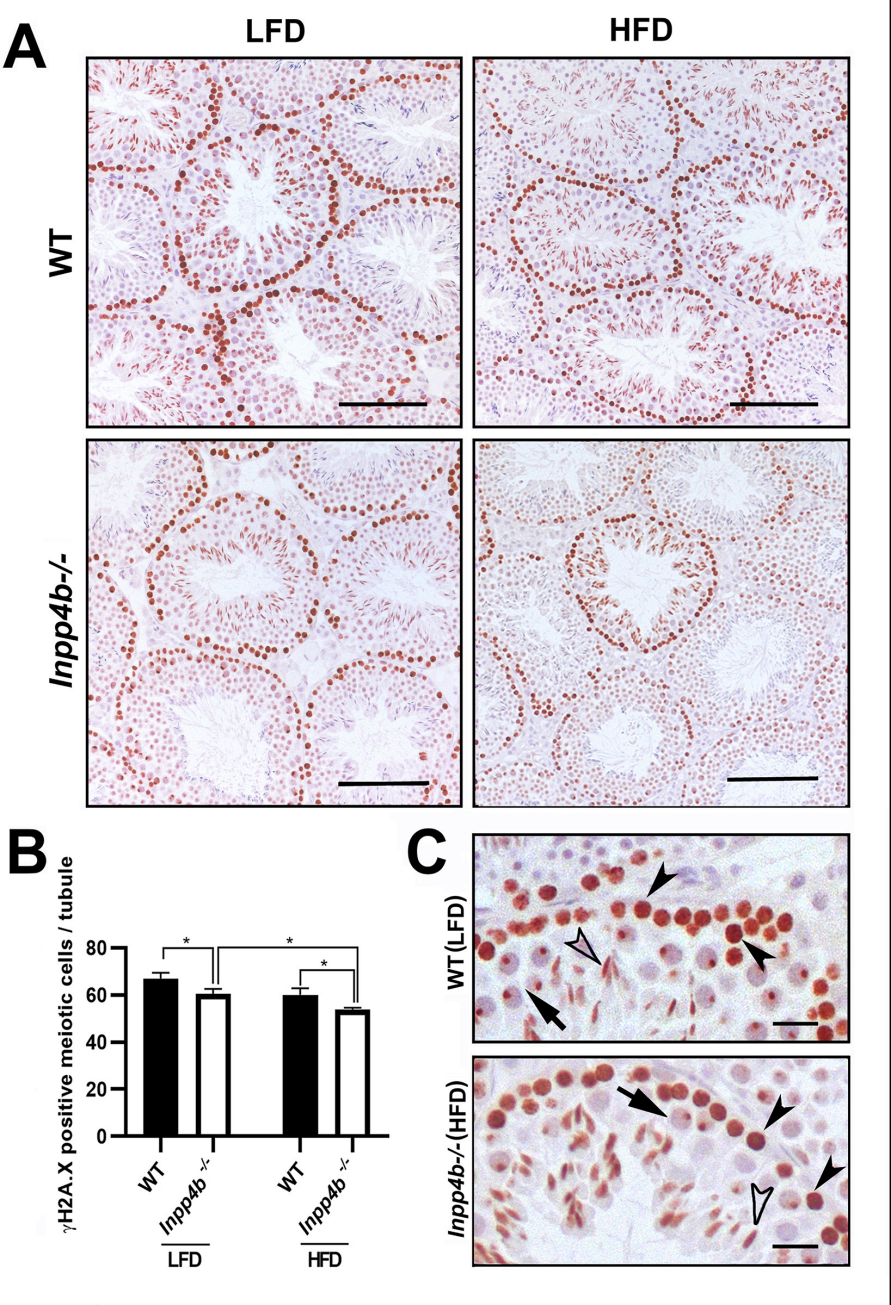
Expression of meiotic marker ϒH2A.X in *Inpp4b*^*-/-*^ testis. A) ϒH2A.X positive cells from the 3-month old WT and *Inpp4b*^**-/-**^ males on LFD or HFD. A representative image from each group is shown. Scale bar represents 200 μm. B) The ϒH2A.X-positive cells (brown staining) at prophase I in stage VIII-XI tubules were counted under 40X objective in at least 10 tubules that appear circular on the slide per animal. *p<0.05; n = 3/group. Data shown as mean ± SEM. Statistical analysis was performed using 2-way ANOVA. C) Magnified sections from WT males on LFD and *Inpp4b*^**-/-**^ males on HFD, pre-leptone to zygotene spermatocytes, pachytene spermatocytes and elongated spermatids showed by black arrowheads, arrows and white arrowheads respectively. Scale bar is 20 μm.

We next analyzed the expression of a meiosis marker, phosphorylated form of histone 2A.X (γH2A.X), which marks the double strand breaks in preleptotene through zygotene spermatocytes and sex bodies in pachytene spermatocytes. It is highly expressed in B type spermatogonia, primary spermatocytes from preleptotene to pachytene stages and elongated spermatids [36–39] (Fig 9A). Based on their position within the seminiferous tubules and cellular morphology at stage VIII-XI, the γH2A.X-positive cells were primary spermatocytes from preleptotene to pachytene spermatocytes, a population of actively dividing cells that determines efficiency of sperm production, and elongated spermatids (Fig 9C). The number of γH2A.X-positive cells were significantly lower in the LFD *Inpp4b*^*-/-*^ group compared to LFD WT controls (p = 0.0178). Consistent with reduced sperm count in HFD *Inpp4b*^*-/-*^ males, there was a significantly lower number of γH2A.X-positive cells in the HFD *Inpp4b*^*-/-*^ group when compared to HFD WT (p = 0.0348) or LFD mutant testes (p = 0.0258) (Fig 9B). Among the prophase I primary spermatocytes, the γH2A.X positive pachytene spermatocyte count was significantly higher in LFD WT males (37.63 cells per tubule) than in the LFD *Inpp4b*^*-/-*^ (31.82 cells/per tubule, p = 0.0135) and HFD *Inpp4b*^*-/-*^ (26.30 cells per tubule, p = 0.0002) groups.

### Expression of cytokines is altered in INPP4B deficient testis

**Fig 10:**
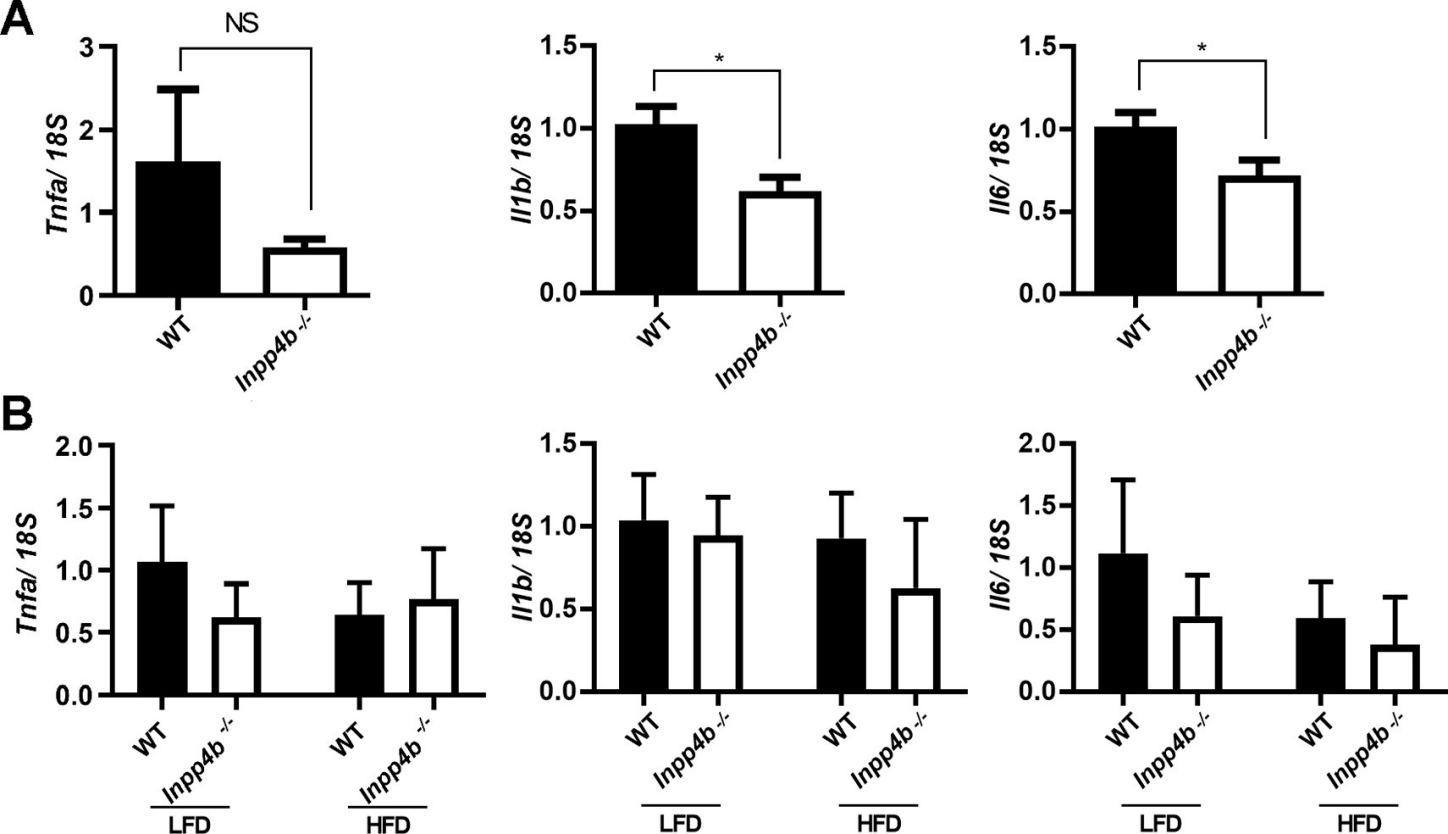
Expression of pro-inflammatory markers in *Inpp4b*^-/-^ testis. A) Reduced expression of *Il1b* and *Il6* in *Inpp4b*^*-*/-^ testes from 4 month-old males on LFD. qRT-PCR data were normalized to the expression of 18S. *p<0.05; n = 8/group. Statistical analysis was performed using Student’s t-test. B) Expression of pro-inflammatory markers in testes from 3-month old WT and *Inpp4b*^*-/-*^ on LFD and HFD. n = 7/group. Analyzed by 2-way ANOVA. Data shown as mean ± SEM.

Cytokines like interleukins (ILs) and tumor necrosis factors (TNFs) have been shown to play an important role in testicular homeostasis and spermatogenesis [40]. In testis, IL1β, IL6 and TNFα are produced by Sertoli, Leydig, and germ cells in a cyclical manner, stimulating Sertoli cell function, spermatogenesis, and steroidogenesis in Leydig cells [41]. We compared the expression of *Il1b*, *Il6* and *Tnfa* in 4-month old WT and *Inpp4b*^*-/-*^ testis on LFD (Fig 10A) and in 3-month old WT and *Inpp4b*^*-/-*^ testes in mice on LFD and HFD (Fig 10B). At three months no significant difference was observed in *Il1b* and *Il6* expression, however, *Il1b* and *Il6* expression was significantly higher in the WT than in *Inpp4b*^*-/-*^ testes in 4-month old males. The expression of *Tnfa* showed no significant difference between any groups (Fig 10).

## Discussion

Recent data demonstrated that PIP signaling plays an important role in spermatogenesis and germ cell maintenance [9]. The involvement of members of this pathway in testicular functions was revealed through analysis of loss-of-function mutants. It was shown the PTEN/ PI3K/Akt pathway is important in controlling the proliferation and division of spermatogonial stem cells in mouse testis. Disruption of this signaling through knockout of the key genes in this pathway leads to the loss of spermatogonial cells and infertility in males [9, 42, 43]. Studies performed on Drosophila indicate that PI(4,5)P2 and PI(4)P are central regulators in germ cell meiosis and spermiogenesis, however the role of this pathway in mammals is less clear.

In this study we analyzed the expression and function of INPP4B in testes. INPP4B antagonizes the PI3K-AKT/PKB signaling pathway by dephosphorylating phosphoinositides and thereby modulating cell cycle progression and cell survival. We have shown that during spermatogenesis, in mice and men, INPP4B expression significantly increases at the round spermatid stage and continues into the mature sperm stage. Infertile men with no spermatids and cryptorchid male mice with spermatogenesis arrest at the spermatogonial-spermatocyte stage both had significantly lower levels of testicular INPP4B expression. Analysis of testicular phenotype in *Inpp4b*^*-/-*^ males showed reduced testis weight, lower sperm count, increased apoptosis rate, and lower LH concentrations. The expression of certain enzymes mediating early stages of androgen synthesis was also reduced in mutant testes. Importantly, some of these abnormalities were exacerbated in animals maintained on a high fat diet, suggesting that INPP4B plays a role in male germ cell differentiation.

In both mice and men, high fat diet correlates with impaired intratesticular signaling and spermatogenesis [44, 45]. PKC pathway dysregulation caused by diet with high fat content leads to disruption in several pathways such as lipid metabolism and reactive oxygen species (ROS) formation, which are important for normal testis functions [46, 47].The synergistic stimulatory effect of *Inpp4b* loss and HFD on the PKC pathway might have a negative impact on testosterone metabolism and spermatogenesis. Expression of steroidogenic enzyme *Cyp17a1* and luteinizing hormone receptor *Lhcgr* were decreased in the HFD groups which, in combination with the decrease of *Srd5a1*, led to a reduction in sperm count in *Inpp4b*^*-/-*^ mice when compared to the LFD group. Increased germ cell apoptosis and the reduction of expression of meiotic marker γH2A.X in the HFD mutant group support this conclusion. It should be pointed out that the relatively modest effects of the HFD in younger males might be due to the resistance of the FVB mouse strain to HFD induced obesity [48].

Analysis of previously reported gene expression datasets revealed a high level of expression of INPP4B in human and mouse testes [15, 17]. In cryptorchid mice, in which the testes are devoid of germ cells beyond the early spermatocyte stage, there was a low level of *Inpp4b* gene expression. The same was true when we analyzed previously published data on gene expression in infertile men with no detectable spermatids [26]. The advance of single cell RNA sequencing allowed us to map the highest *INPP4B* expression to postmeiotic germ cells beginning from the round spermatids; the conclusion was also supported by IHC analysis in mouse testis. Consistently, the genes positively correlated with expression of *INPP4B* were associated with pathways activated during spermatogenesis, such as spermatid differentiation, flagellum formation, and fertilization. The question arises as to whether INPP4B plays any functional role in these processes or whether it is just a marker for specific stages of spermatogenesis. Our data suggest that the former might be true. INPP4B mainly catalyzes the hydrolysis of the phosphate located in the 4^th^ position of inositol ring of PI(3,4)P2, PI(4,5)P2, and inositol 1, 3, 4-trisphosphate. Experiments in Drosophila clearly show that PI4P cell signaling in germ cells has the same pattern: it is most prominent in spermatocyte through spermatid stages of differentiation, showing modest effects on the premeiotic population. Germline stem cells are affected by the PI3K pathway, mediated by insulin receptor and FOXO transcription factors. All these pathways are nutritionally regulated [9]. Activation of the PI3K pathway (especially isoform PI3KDN) in somatic cyst cells improves the transition from germline stem cells to spermatocytes [49]. PI4P and PI(4,5)P2 are regulators of meiotic cytokinesis in Drosophila spermatocytes [9]. Deletion of *four wheel drive* (*fwd)* encoding Drosophila PI 4-kinase IIIβ (PI4KIIIβ) [50], Class I PI transfer protein (PITP) [51, 52], trafficking factors such as GOLPH3, a Golgi PI4P-binding protein [52], and other members of PIP pathway all lead to abnormal cytokinesis. Involvement of the homologous genes in spermatogenesis in mice is less clear. Here we showed that INPP4B is highly expressed in postmeiotic germ cells during spermatogenesis in mice and men, and the deletion of this gene in mice leads to a decrease in mature sperm. However, the mild effect of *Inpp4b* deficiency is likely due to compensation by other members of the pathway, by modifier genes, or the relatively young age of the analyzed mice.

The deletion of another member of the INPP family, INPP5B, causes male infertility in mice due to a reduced sperm count, motility, and fertilization defects [53]. Interestingly, that backcrossing of the mutant allele on FVB/N inbred background, the same genetic background as in our mice, partially rescued mutant male infertility, suggesting the existence of genetic modifier gene(s) in this mouse line. Conditional deletion of *Inpp5b* in germ cells resulted in normal male fertility [53], indicating that the abnormalities in mutant sperm function and maturation were not due to a deletion of the gene in germ cells. Further analysis of *Inpp5b*^*-/-*^ mutant Sertoli cells revealed the appearance of abnormal vacuoles affecting germ cell adhesion [54]. A similar effect of *Inpp4b* deletion in somatic testicular cells may account for the observed increase of apoptosis in spermatogonial and spermatocyte cells detected in *Inpp4b*^*-/-*^ mutants. One possible explanation of this phenomenon is that it might be related to a decrease in testosterone production in mutants along with the reduced expression of several steroidogenesis genes in mutant testis. The role of INPP4B targets, such as PI3K/Akt, in the survival of Leydig cells and steroidogenesis has been previously demonstrated [55]. Additionally, decreased expression of cytokines such as *Il1b* and *Il6* in mutant Sertoli cells might have caused reduced testosterone production, resulting in impaired spermatogenesis [4]. Ablation of AR signaling in Leydig, Sertoli, or peritubular myoid cells all leads to deficient spermatogenesis [56]. This is also consistent with our findings of prostate hyperplasia in one year old *Inpp4b* mutant males [23]. Thus, it is possible that in addition to a direct effect of *Inpp4b* deletion in germ cells, indirect effects of reduced testosterone and LH signaling in testicular somatic cells may be responsible for the observed phenotype. Further analysis of conditional *Inpp4b* deletion in various testicular cells might define the role of this gene in spermatogenesis.

## Supporting information

S1 Fig Seminiferous tubule measurements in *Inpp4b* ^*-/-*^ mice. H&E stained testis seminiferous tubules of 2-, 3- (LFD and HFD), 4- and 6-month old mice were measured under 20X objective and analyzed with 2-way ANOVA. Data shown as mean ± SEM. n = 3/group.
(TIF)

S1 Raw data (PDF)
