# Supplementary figures and images for: Deletion of inositol polyphosphate 4-phosphatase type-II B affects spermatogenesis in mice
Source: PLoS One. 2020 May 15;15(5):e0233163. doi: 10.1371/journal.pone.0233163 (PMC7228085; doi:10.1371/journal.pone.0233163)

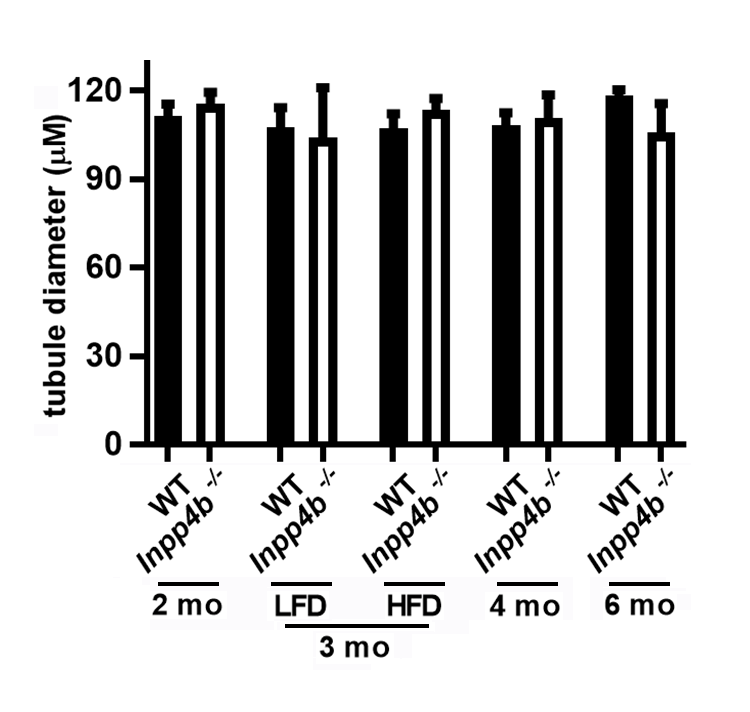

Supplement: S1 Fig — H&E stained testis seminiferous tubules of 2-, 3- (LFD and HFD), 4- and 6-month old mice were measured under 20X objective and analyzed with 2-way ANOVA. Data shown as mean ± SEM. n = 3/group. (TIF) [file pone.0233163.s001.tif]

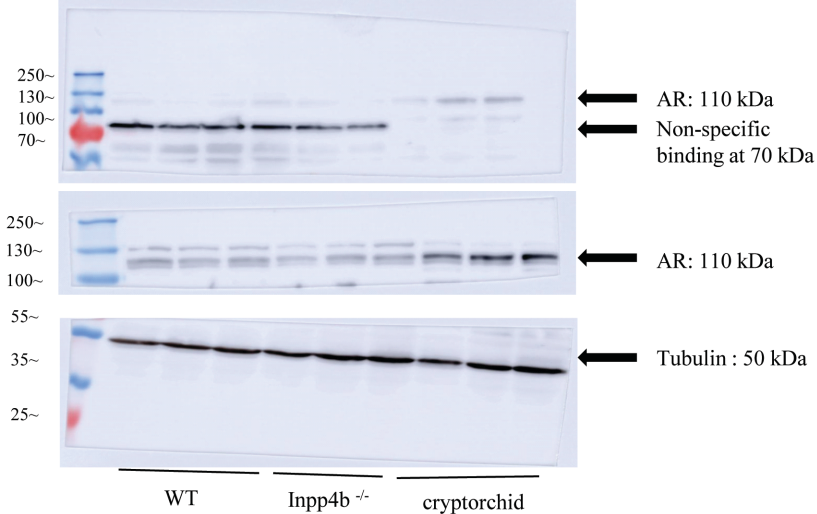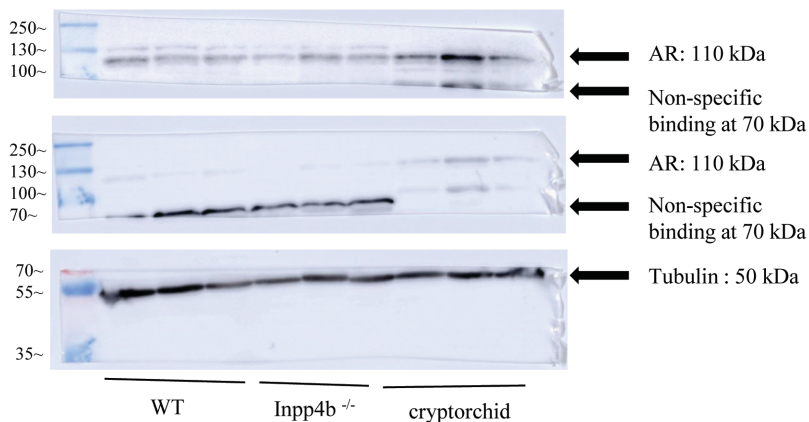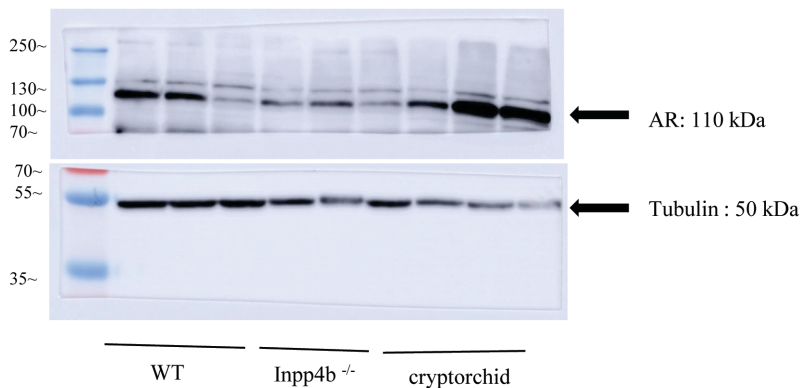

Supplement: S1 Raw data — (PDF) [file pone.0233163.s002.pdf]
